# Evolutionary Dynamics of the Accessory Genome of *Listeria monocytogenes*

**DOI:** 10.1371/journal.pone.0067511

**Published:** 2013-06-25

**Authors:** Henk C. den Bakker, Christopher A. Desjardins, Allison D. Griggs, Joseph E. Peters, Qiandong Zeng, Sarah K. Young, Chinnappa D. Kodira, Chandri Yandava, Theresa A. Hepburn, Brian J. Haas, Bruce W. Birren, Martin Wiedmann

**Affiliations:** 1 Department of Food Science, Cornell University, Ithaca, New York, United States of America; 2 The Broad Institute, Massachusetts, United States of America; 3 Department of Microbiology, Cornell University, Ithaca, New York, United States of America; University of North Dakota School of Medicine and Health Sciences, United States of America

## Abstract

*Listeria monocytogenes*, a foodborne bacterial pathogen, is comprised of four phylogenetic lineages that vary with regard to their serotypes and distribution among sources. In order to characterize lineage-specific genomic diversity within *L. monocytogenes*, we sequenced the genomes of eight strains from several lineages and serotypes, and characterized the accessory genome, which was hypothesized to contribute to phenotypic differences across lineages. The eight *L. monocytogenes* genomes sequenced range in size from 2.85–3.14 Mb, encode 2,822–3,187 genes, and include the first publicly available sequenced representatives of serotypes 1/2c, 3a and 4c. Mapping of the distribution of accessory genes revealed two distinct regions of the *L. monocytogenes* chromosome: an accessory-rich region in the first 65° adjacent to the origin of replication and a more stable region in the remaining 295°. This pattern of genome organization is distinct from that of related bacteria *Staphylococcus aureus* and *Bacillus cereus*. The accessory genome of all lineages is enriched for cell surface-related genes and phosphotransferase systems, and transcriptional regulators, highlighting the selective pressures faced by contemporary strains from their hosts, other microbes, and their environment. Phylogenetic analysis of O-antigen genes and gene clusters predicts that serotype 4 was ancestral in *L. monocytogenes* and serotype 1/2 associated gene clusters were putatively introduced through horizontal gene transfer in the ancestral population of *L. monocytogenes* lineage I and II.

## Introduction

In this study we focus on the evolution and dynamics of the accessory genome of the foodborne pathogen *Listeria monocytogenes*. *L. monocytogenes* is a saprotrophic Firmicute, which can be commonly found in the environment. In case of an (usually foodborne) infection of a susceptible host, it switches from a saprotrophic to an intracellular pathogenic lifestyle and can cause a severe systemic infection termed listeriosis [1]. Current population genetic and phylogenetic data show that *L. monocytogenes* can be subdivided into four phylogenetic lineages, designated Lineage I, II, III and IV, which seem to differ in ecology, recombination rates and genomic content [2]. Lineage I strains seem to be overrepresented among human clinical cases in many countries, while lineage II strains are common in foods and seem to be widespread in natural and farm environments [2]. Lineage III and IV strains are rare among human clinical cases and in foods compared to strains of the other lineages and have been associated with animal clinical cases [3].

Traditional subtyping of *L. monocytogenes* has relied on serotyping [4]. *L. monocytogenes* serotypes are predominantly determined by somatic (O-) antigens, with 12 recognized O-antigens, which are highly variable between serotypes. Flagellar (H-) antigens are less abundant (only four antigens in *L. monocytogenes*) and are conserved in the majority of the *L. monocytogenes* serotypes [5]. Serotypes 4b and 1/2b are the dominant serotypes in lineage I, while serotypes 1/2a and 3a are the most common serotypes in lineage II [2], [6]. Lineages III and IV contain the serotypes 4a, 4b and 4c [3]. O-antigenic variation is correlated to the biochemistry of the wall teichoic acids, components of the cell wall, which are exposed to the external milieu [7]–[9]. In particular the decoration of the wall teichoic acids seems to be correlated to serotype, ranging from no decoration in serotype 7, rhamnose based decorations in serotypes 1/2, and glucose and galactose based decorations in variants of serotypes 4, 5 and 6 [7], [10]. To our knowledge, only rhamnose has so far been proven experimentally to be a major antigenic determinant, for serotype 1/2a [9]. The major antigenic determinants for the other serotypes still have to be experimentally confirmed. The prevailing hypothesis based on population genetic research of *L. monocytogenes* has been that the most recent common ancestor of *L. monocytogenes* had a 1/2b serotype, and that the 4b serotype arose only recently from a 1/2b ancestor [6], [11]. The number of serotypes recognized within *L. monocytogenes* is very small as compared to pathogens such as *Salmonella enterica*, which has more than 2,600 distinct serotypes [12], [13]. This suggests that cell surface-related proteins responsible for antigen variation in *L. monocytogenes* may be under less diversifying selection as compared to other pathogens.

Comparative genomic research on *L. monocytogenes* has previously focused on pan/core genome size estimates and the role of recombination and positive selection in the evolution of the core genome [14]–[16]. The pan-genome (the collection of all genes) of a given bacterial taxonomical unit (TU; usually a species or genus) can be subdivided into the accessory genome (the collection of genes found in a subset of strains but not all strains of the TU) and the core genome (the collection of genes found among all strains of the TU). The core genome can be used to identify the specific genomic characteristics of a given TU, while the size, content and dynamics of the accessory genome can be an indicator of the plasticity or adaptability of a given TU [17]. The accessory genome of different populations within a bacterial species can differ significantly due to selective pressures experienced in different environments [18]. Therefore knowledge of the content and dynamics of the accessory genome of individual populations within a species may elucidate the kind of selective pressures experienced by these populations and increase our understanding of the ecology of a species. Here, we sequenced the genomes of 8 strains of *L. monocytogenes*, including representatives of lineages I, II, and III, and previously unsequenced serotypes 3a and 1/2c. We used these data to characterize the evolutionary dynamics of the accessory genome of *L. monocytogenes* to gain a better understanding of the genome organization of this pathogen and further focus on the evolution of O-antigen associated genes.

## Materials and Methods

### Bacterial Strains and Genome Sequencing

Bacterial strains used in this analysis and basic assembly information of each strain can be found in Table 1. In addition to the newly sequenced genomes presented in this paper we added representative published genomes [19]–[25] to the analysis. Sanger sequences were generated from three whole genome shotgun sequencing libraries for each strain (two plasmid libraries (4 kb and 10 kb inserts) and a Fosmid library (40 kb inserts)), using ABI 3730 machines as described previously [26]. The remaining sequences were generated using 454 [27] and Illumina [28] technology. Genome sequences of strains J0161, 10403S, FSL R2-561, and Finland 1998 were assembled with HybridAssemble from the September 2008 version of the Arachne assembly package [29] using both Sanger and 454 sequences. Assemblies for the other strains were created with Newbler 1.1.03.19 (http://454.com/products/analysis-software/index.asp) using 454 data and were then improved using SolexaPoly (from the September 2008 Arachne assembly package), which uses Illumina sequence data to correct 454 errors.

**Table 1:** Genomes and strains used for analyses.

Strain	Sero-type	Line-age	Source	Sequencing center	Coverage & Status	Accession
*L. monocytogenes*						
10403S	1/2a	II	Streptomycin resistant lab strain, USA	Broad Institute v6	48×finished	AARZ00000000.3
Finland1998	3a	II	Butter associated outbreak, Finland	Broad Institute v3	48×finished	AART00000000.2
FSL R2-561	1/2c	II	Human sporadic case, England	Broad Institute v2	48×finished	AARS00000000.2
J0161	1/2a	II	Human clinical case, USA	Broad Institute v8^2^	29×finished	AARW00000000.3
F6900	1/2a	II	Human clinical case, USA	Broad Institute v3^2^	26×draft	AARU00000000.2
FSL J1-194	1/2b	I	Human sporadic case, USA	Broad Institute v3^2^	22×draft	AARJ00000000.2
FSL J2-071	4c	III	Bovine clinical case, USA	Broad Institute v5	21×draft	AARN00000000.4
FSL N1-017	-1	I	Food processing environment, USA	Broad Institute v5	22×draft	AARP00000000.4
FSL R2-503	1/2b	I	Human clinical case, USA	Broad Institute v3	35×draft	AARR00000000.2
FSL N3-165	1/2a	II	Farm environment, USA	Broad Institute v3	22×draft	AARQ00000000.2
Aureli1997(HPB2262)	4b	I	Human clinical case, Italy	Broad Institute v3	20×draft	AARL00000000.2
J2818	1/2a	II	Food, outbreak related, USA	Broad Institute v3^2^	24×draft	AARX00000000.2
EGD-e	1/2a	II	Lab strain, origin unknown	Institut Pasteur	finished^3^	NC_003210
H7858	4b	I	Food, outbreak related, USA	TIGR	8×draft	AADR00000000
F6854	1/2a	II	Food (Turkey Franks), USA	TIGR	8×draft	AADQ00000000
F2365	4b	I	Food, USA	TIGR	8×finished	AE017262
CLIP80459	4b	I	Human clinical case, France	Institut Pasteur	finished^3^	NC_012488
08_5578	1/2a	II	Human clinical case, Canada	Public Health Agency of Canada	40×finished	CP001602
08_5923	1/2a	II	Human clinical case, Canada	Public Health Agency of Canada	36×finished	CP001604
HCC23	4a	III	Healthy catfish, USA	Mississippi State University	15×finished	NC_011660
FSL J1-208	4a	IV	Caprine clinical case, outbreak related	Cornell University/Broad Institute	200×draft	AEIS01000000
*L. marthii*						
FSL S4-120^4^	6a		Forest soil, NY, USA, 2001	Cornell University/Life Technologies	200×draft	CM001047.1
*L. innocua*						
CLIP11262	6a		Wild type lab strain	Institut Pasteur	finished^3^	NC_003212
FSL S4-378^4^	4ab		Puddle of water, NY, USA, 2002	Cornell University/Life Technologies	200×draft	NZ_CM001048.1
FSL J1-023^4^	4b		Obtained from Qualicon	Cornell University/Life Technologies	200×draft	NZ_CM001049.1
*L. welshimeri*						
SLCC5334	6b		Decaying vegetation, USA	Justus-Liebig-University	6.4×finished	AM263198
*L. seeligeri*						
SLCC3954	1/2b		Soil, Germany	Justus-Liebig-University	7×finished	NC_013891
FSL N1-067^4^	7		Food processing plant	Cornell University/Life Technologies	200×draft	NZ_CM001051.1
FSL S4-171^4^	4c		Urban environment, NY, USA, 2001	Cornell University/Life Technologies	200×draft	NZ_CM001052.1
*L. ivanovii*						
PAM 55^4^	5		Sheep, Spain	Institut Pasteur	8×finished	NC_016011.1
ATCC 49954^4^	5		Food, France	Cornell University/Life Technologies	200×draft	NZ_CM001050.1

1 While FSL N1-017 has been reported to be serotype 4b (http://www.broadinstitute.org/annotation/genome/listeria_group/GenomeDescriptions.html), gene content analysis suggests that it should be serotype 1/2b.

2 These genomes were previously sequenced [56], but here the assemblies were improved and the genomes were re-annotated.

3 Coverage information for these genome sequences could not be determined.

4 Genome sequences only used in O-antigen related analyses. Serotypes for these strains, with the exception of *L. ivanovii*, were newly determined in this study.

### Serotyping

Classical serotyping was performed for a select number of isolates of *Listeria innocua* (FSL S4-378, FSL J1-023), *Listeria seeligeri* (FSL N1-067, FSL S4-171) and *L. marthii* (FSL S4-120), using antisera from Denka Seiken (Denka Seiken Co Ltd, Tokyo, Japan).

### Annotation

Protein-coding genes were predicted using a combination of ab initio, synteny-based, and homology-based gene prediction methods. For ab initio gene predictions, ORFs were predicted using Glimmer3 with default parameters [30], MetaGene with default parameters [31], and GeneMark trained with the 500 longest ORFs predicted by Glimmer3 [32]. Synteny-based gene prediction was conducted as previously described [33], using default parameters for both Nucmer [34] and LAGAN [35] alignments and strains EGD-e and F2365 as reference genomes. In regions without ab initio or synteny-based gene models, homology-based gene models were constructed from BLAST hits to the non-redundant protein database with an e-value cutoff of 1×10^−10^. Gene product names were assigned based on BLAST hits to the UniRef90 database and hmmer hits to TIGRfam and PFAM, and every gene was assigned a unique locus number of the form xxxG_#####. Ribosomal RNAs were identified with RNAmmer [36], tRNA features were identified using tRNAScan [37], and other non-coding features were identified with RFAM [38].

### Gene Ontology and Enrichment Analysis

Overrepresentation (enrichment) of certain Gene Ontology (GO) categories in the core versus the accessory genome and in the region around the chromosomal origin of replication was tested using a Bonferroni corrected Fisher’s exact test. Gene Ontology terms were assigned to each gene using Blast2GO [39] with an e-value cutoff of 1×10^−10^.

### Gene Clustering and Evolutionary Analyses

Orthology assignment was performed with OrthoMCL 1 [40] with a Markov inflation index of 1.5 and a maximum e-value of 1e-5, using the default parameter settings. We defined core genes as those present in all 10 finished genomes and accessory genes as those missing from at least 1 finished genome. Sequences of these clusters were aligned using MUSCLE [41], poorly aligned regions were trimmed using trimAl under default settings [42], and individual gene phylogenies were estimated using FastTree [43]. We then calculated dN/dS for each cluster using the CODEML program of the PAML package (version 4.4) using the model of a single omega for all branches [44]. To generate an organismal phylogeny we concatenated alignments of the 2,086 genes that were present as single copies in all genomes, and estimated a phylogeny using the GTRMIX model in RAxML [45]. The tree was made ultrametric using PathD8 [46] for ease of visualization.

### Insertion/Deletion Hot Spot Analyses

Insertion/deletion hotspot maps were created as described in Touchon et al. [47]. In short, genes present in all strains (single copy core genes) were plotted on the X-axis, while genes that were present in the insertion/deletion regions (the accessory genome) were plotted on the y-axis at the position relative to the adjacent core genome genes. To test if two groups (*L. monocytogenes* vs *Staphylococcus aureus*) have different accessory gene distributions across the chromosome, we plotted the cumulative distribution of accessory genes over the chromosome. Positions of the accessory genes on the genome were transformed to degrees (following the formula given in [48]) to allow comparisons of the distribution of the accessory genome among genomes, even in the presence of frequent genome rearrangements. Prophage related genes were excluded from this analysis. We then identified the degree position that divided the genome into two regions and maximized the χ^2^ value of the difference in the distributions of core and accessory genes.

### Evolution of Genes Associated with O-antigen Variation

Genes with phylogenetic histories discordant with the major lineage divisions were identified using a previously described method [49]. Briefly, each gene was assigned a value based on its position within the phylogenetic tree of its orthologs within *L. monocytogenes*. These values were mapped to a gradient ranging from dark red (groups solely with lineage I, III, and IV) to dark blue (groups solely with lineage II). Then each gene of each genome was plotted by color against the reference genome of strain F2365, using Circos [50]. Nucleotide sequences of genes from discordant regions in *L. monocytogenes*, along with genes from additional *Listeria* species (*L. innocua* CLIP11262 serotype 6a, FSL S4-378 serotype 4ab, FSL J1-023 serotype 4b; *L. seeligeri* SLCC3954 serotype 1/2b, FSL S4-171 serotype 4c, FSL N1-067 serotype 7; *L. marthii* FSL S4-120 6a; *L. ivanovii* subsp. *ivanovii* PAM and *L. ivanovii* subsp. *londoniensis*; both serotype 5) were aligned using MUSCLE version 3.8.31 [41]. Phylogenetic trees were inferred from these alignments using the maximum likelihood criterion in PHYML version 3 [51], with 100 bootstrap replicates. Maximum likelihood trees were inspected and categorized into two groups; (i) trees primarily clustering according to the organismal tree (that is the phylogenetic relationships are congruent to the inter- and intraspecific phylogenies of *Listeria* as inferred in [52]) and (ii) trees that cluster according to serotype. To reconcile individual gene trees with the organismal tree, AnGST [53] (http://almlab.mit.edu/angst/) and Mowgli [54] (http://www.atgc-montpellier.fr/Mowgli/) were used. AnGST was run using the event penalties recommended by the authors of the software (horizontal gene transfer: 3, gene duplication: 2, gene loss: 1, and speciation: 0), Mowgli was ran using the default parameters, with the exception that nearest neighbor editing was allowed for branches with a bootstrap support <60.

Recombination Analysis Tool (RAT: [55]) was used to detect putative recombination breakpoints in gene clusters.

## Results

### *L. monocytogenes* Genomes are Highly Conserved

We sequenced the genomes of eight strains of *L. monocytogenes* (Table 1), yielding three finished (single scaffold) and five high-quality draft (coverage ≥20X, multiple scaffolds) genomes. Furthermore, we generated improved assemblies for four previously published genomes [56], resulting in one additional finished and three high-quality draft genomes. All genomes were annotated (see methods) and resulting statistics are shown in Table 2. In Table 1, we compare these genomes to an additional six finished and three annotated draft genomes already available in Genbank. Genome size in *L. monocytogenes* genomes is tightly conserved, ranging from the 2.74 Mb genome of FSL J1-208 to the 3.14 Mb genome of FSL N1-017, and is not correlated with lineage membership. As expected, the largest and smallest genomes also had the fewest and most genes, 2,765 and 3,187, respectively.

**Table 2:** Genome statistics of *L. monocytogenes* genome sequences used in this study.

Strain	No. of Scaffolds	Genome Size (Mb)	No. of Genes	Contig N50 (kb)^1^	Q40 bases (%)^2^	No. of Accessory Genes	No. of alignable Non-Phage Acc. Genes	No. of Unique Genes
10403S	1	2.90	2828	N/A	99.9	389	336	12
F6900	23	2.97	3005	524	99.9	567	439	5
Finland1998	1	2.87	2762	N/A	98.8	323	323	11
FSL J1-194	30	2.99	3013	354	99.8	577	430	32
FSL J2-071	53	2.85	2822	145	99.3	398	334	72
FSL N1-017	79	3.14	3187	92	99.5	753	448	134
FSL R2-503	55	2.99	3027	173	99.5	592	426	47
FSL N3-165	39	2.88	2890	147	99.7	451	441	40
FSL R2-561	1	2.97	2910	N/A	99.7	471	347	29
Aureli1997 (HPB2262)	79	2.99	3053	143	99.6	616	434	71
J0161	1	3.00	2973	N/A	99.9	534	403	3
J2818	24	2.97	3084	489	99.8	646	503	38
EGDe	1	2.94	2864	N/A	–	425	344	8
H7858	181	2.97	3111	N/A	–	700	530	227
F6854	133	2.95	2967	N/A	–	568	455	67
F2365	1	2.91	2821	N/A	–	382	382	31
CLIP80459	1	2.91	2766	N/A	–	327	327	12
08_5578	1	3.03	3010	N/A	–	571	404	33
08_5923	1	3.00	2966	N/A	–	527	406	5
HCC23	1	2.98	2974	N/A	–	535	338	127
FSL J1-208	8	2.74	2765	527	–	401	401	96

Contig N50 values are given for draft (unfinished) genomes assembled here, while the percent Q40 bases is given for all genomes assembled here.

1 N/A = not available, because the genome sequence is closed.

2 Percentage Q40 bases is only given for genome sequences newly presented in this publication.

OrthoMCL [40] was used to identify clusters of orthologous genes across all *Listeria* genomes. We identified 2,439 *L. monocytogenes* core genes present in all 10 completely sequenced genomes, similar to previously estimated size (between 2,330 and 2,465 genes) of the core genome of *L. monocytogenes* [26], [62]. The accessory genome represents a small fraction of *L. monocytogenes* gene content relative to the core genomes (12–23%). Therefore, while there is substantial variation in the size of the accessory genome (which ranges from 323 to 753 genes per strain), genome size and the total number of genes are highly conserved across *L. monocytogenes* strains. Variation in accessory genome size can be due to many factors, including biological factors such as the presence/absence of various prophages in the *L. monocytogenes* genomes (as previously shown in Den Bakker et al. [24]) or artifacts such as completeness of the genome assemblies.

### The First 65 Degrees Adjacent to the Origin of Replication of *L. monocytogenes* are Significantly Enriched for Accessory Genes

**Figure 1:**
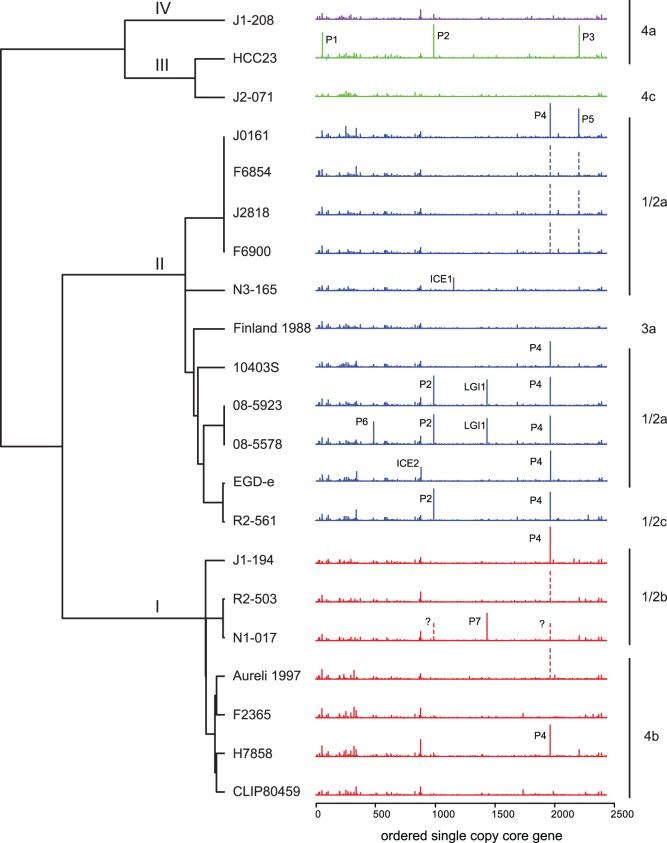
***L. monocytogenes*** phylogenetic tree and accessory genome distribution plots. Plots show the number of accessory genes in between each core gene as ordered in the reference strain EGDe. Insertion sites of prophages (P), integrated conjugative elements (ICE), and *Listeria* genomic islands (LGI) as detailed in Table 4 are indicated above each accessory genome distribution plot. Vertical dotted lines with a question mark indicate prophages, which are not assembled in a single contiguous piece, but are hypothesized to be present in the location based on presence of the appropriate phage genes in unalignable fraction of the assembly. Plots are colored by lineage: I, red, II, blue, III, green, IV, purple. Serotypes are shown to the right of each plot. The phylogenetic tree is based on a maximum likelihood analysis of the concatenated alignments of 2,086 core genes.

Utilizing the 2,086 genes identified as orthologous across all *Listeria* species, we constructed a phylogeny *of L. monocytogenes* genomes rooted with outgroup genomes of the closely related species *L. innocua*, *L. welshimeri*, and *L. seeligeri* (Fig. 1, outgroups not shown). This phylogeny agrees with previous phylogenetic analyses [52], [57] and divides *L. monocytogenes* into its four major lineages. To examine the positioning of the accessory genes along *L. monocytogenes* genomes in a phylogenetic context, the number of accessory genes between each core gene was plotted for each genome (Fig. 1). Positioning of accessory gene clusters is conserved across *L. monocytogenes* genomes, as was observed by Touchon et al. in *E. coli* [47]. The distribution of accessory gene clusters over the chromosome in *L. monocytogenes* seems to differ from that of *E. coli* in that in *L. monocytogenes* there is a high concentration of these accessory gene clusters close to the origin of replication. This is particularly true in the region spanning the first approximately 500 Kb of the chromosome. While this paper was under review, Kuenne et al. [58] published an analysis of accessory gene distribution using a largely non-overlapping set of *L. monocytogenes* strains, and also found genes clustered into insertion-deletion hotspots. Independent confirmation of these insertion-deletion hotspots in different sets of genomes by Kuenne et al. [58] and this study show that these hotspots are highly conserved among *L. monocytogenes* strains. Concentration of hotspots to the right of the origin of replication is also supported by Kuenne et al. [58], who found eight out of nine insertion deletion hotspots identified in their study to be positioned in the right replichore. In our work, however, we noted that genomic change is not restricted to these hotspots, but that the whole region of the chromosome adjacent to the origin of DNA replication is prone to insertion and deletion events (see below) and can be considered a ‘hot region’.

**Figure 2:**
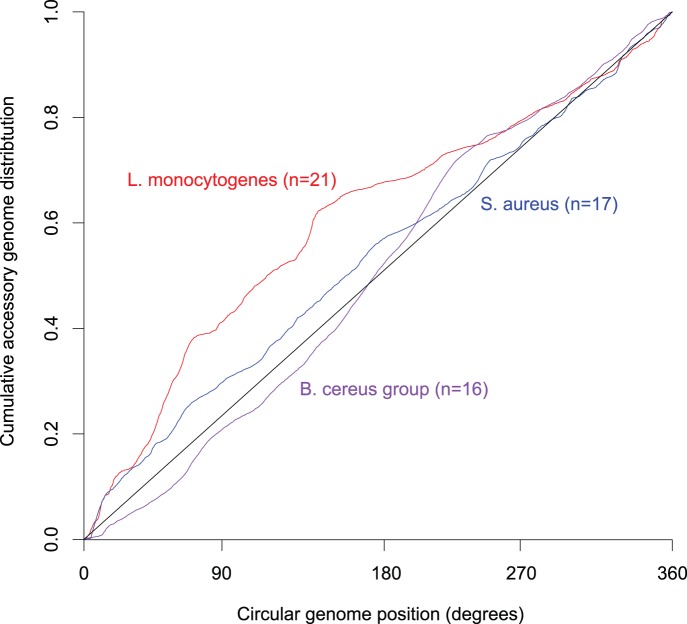
Cumulative distribution of the accessory genome throughout the chromosome in *L. monocytogenes* (n = 21), *Staphylococcus aureus* (n = 17) and strains of the *Bacillus cereus* group (n = 16). The circular genome position starts at the origin of replication, which is at 0 degrees.

To test if this distribution is uniquely found in *L. monocytogenes* we plotted the cumulative distribution of accessory genes along the chromosome for *L. monocytogenes*, and the phylogenetically closely related species *Staphylococcus aureus* and the *Bacillus cereus* group, with the exclusion of prophage regions. *L. monocytogenes* shows a highly unequal distribution with 38% of the accessory genes found within the first 65° (approximately a 0.5 Mb region) from the origin of replication (χ^2^ = 2411, p<0.0001), while the accessory genomes of *S. aureus* and the *B. cereus* group are more evenly distributed over the chromosome (Fig. 2). The distributions of accessory genes in *S. aureus* and *B. cereus* were significantly different from that of *L. monocytogenes* (*P*<0.0001, Kruskal-Wallis test), confirming the uniqueness of the pattern found in *L. monocytogenes.*

To evaluate whether the strength of selection differs between the different regions of the genome and between core and accessory genes, we calculated dN/dS for all genes shared by at least two *L. monocytogenes* strains. As expected, we found that genes in the accessory genome are less selectively constrained than those in the core genome (median dN/dS = 0.131 and 0.036, respectively, p<0.001, Wilcoxon test). However we also found that core genes in the first 65° of the genome experience significantly less purifying selection than core genes in the last 295° of the genome (median dN/dS = 0.045 and 0.035, respectively, p<0.001, Wilcoxon test). The same pattern was also found for accessory genes (median dN/dS = 0.133 and 0.128, respectively, p = 0.003, Wilcoxon test). This suggests that irrespective of designation as a core or accessory, genes in the first 65° of the genome are more rapidly evolving than those in the last 295°.

We also found differences in the length of intergenic regions. Intergenic regions in the first 65° of the genome are significantly longer than intergenic regions in the last 295° of the genome (p<0.0001, Wilcoxon test). This difference in intergenic length distributions is the result of comparably long regions between neighboring accessory and core genes (median length 85 bp); intergenic regions between accessory and core genes are significantly more common in the first 65° relative to the last 295° (p<0.0001, chi-square test) of the chromosome. Interestingly, the core-core intergenic regions (median length = 45 bp) were found to be significantly longer than accessory-accessory intergenic regions (median length = 24 bp; p<0.0001, Wilcoxon test).

### The Accessory Genome of *L. monocytogenes* is Enriched for Phosphotransferase Systems, Cell Surface Genes, and Prophages

Eight functional categories were found significantly overrepresented in the accessory genome of *L. monocytogenes* and were represented by more than 100 genes in each category (Table 3). These categories relate to four broad classes of genes: (i) phosphotransferase system (PTS) components (involved in sugar transport), (ii) cell wall components, (iii) transcriptional regulators (represented by the sequence-specific DNA binding term), and (iv) mobile elements (represented by the DNA integration term). The enrichment for mobile elements is likely reflective of the numerous large prophages that are unequally distributed across the different *L. monocytogenes* strains (Fig. 1; Table 4). The over-representation of genes corresponding to the remaining three categories likely represents a response to the diverse environmental pressures faced by *L. monocytogenes*.

**Table 3:** Top 25 most abundant Gene Ontology (GO) terms which are significantly enriched in the accessory genome versus the core genome of *Listeria monocytogenes*.

Original GO term	No. of genes in		P value	GO description
	Core genome	Accessory genome		
GO:0009401	1690	411	2.04E-45	phosphoenolpyruvate-dependent sugar phosphotransferase system
GO:0008982	1235	335	1.10E-44	protein-N(PI)-phosphohistidine-sugar phosphotransferase activity
GO:0005351	1020	333	1.35E-59	sugar:hydrogen symporter activity
GO:0009986	302	246	2.81E-103	cell surface
GO:0005618	363	233	1.25E-82	cell wall
GO:0043565	533	150	6.87E-20	sequence-specific DNA binding
GO:0009273	178	124	1.52E-45	peptidoglycan-based cell wall biogenesis
GO:0015074	42	101	4.78E-67	DNA integration
GO:0005576	169	70	4.30E-15	extracellular region
GO:0004802	0	68	1.48E-67	transketolase activity
GO:0005518	42	64	2.42E-35	collagen binding
GO:0005529	250	60	1.53E-04	sugar binding
GO:0004803	20	53	3.15E-35	transposase activity
GO:0006313	62	53	2.95E-21	transposition, DNA-mediated
GO:0004351	0	51	5.75E-50	glutamate decarboxylase activity
GO:0006536	42	51	5.63E-25	glutamate metabolic process
GO:0015755	104	48	3.86E-11	fructose transport
GO:0015573	125	45	2.12E-07	beta-glucoside transmembrane transporter activity
GO:0008861	0	42	1.15E-40	formate C-acetyltransferase activity
GO:0000150	0	41	1.24E-39	recombinase activity
GO:0008706	42	40	1.04E-16	6-phospho-beta-glucosidase activity
GO:0043624	126	40	4.22E-05	cellular protein complex disassembly
GO:0047632	0	38	1.56E-36	agmatine deiminase activity
GO:0006306	21	36	5.30E-20	DNA methylation
GO:0006323	42	35	3.70E-13	DNA packaging

P values were calculated using Fisher’s exact test and Bonferroni corrected.

**Table 4:** Overview of prophage and Inserted Conjugative Elements (ICE) insertion sites in *L. monocytogenes*.

Mobile element integration site in Fig. 1	Type of element	gene closest to integration site (attC)	Insertion site occupied in^1^:
ICE1	ICE	FlaR (lmo1412)	FSL N3-165
ICE2	Integrated conjugative element (ICE)	guaA (lmo1096)	EGD-e (tn916-like: ICELm1)
LGI1	Genomic Island	RNA methyltransferase, TrmA family-Fosfomycin resistance protein FosX	08-5578, 08-5923
P1	Prophage	tRNA-Lys	L99, HCC23 (PSA-like)
P2	Prophage	tRNA-Arg	08-5578, 08-5923, FSL R2-561, FSL N1-017 (B025-like)
P3	Prophage	ribosomal protein S9	L99, HCC23 (A500-like)
P4	Prophage	ComK	J0161, F6854, J2818, F6900, 10403S, 08-5578, 08-5923, EGD-e, FSL R2-561, FSL J1-194, FSL R2-503, FSL N1-017.Aureli 1997, H7858 (A118-like)
P5	Prophage	tRNA-Thr-4	J0161, F6854, J2818, F6900 (A118-like)
P6	Prophage	tRNA-Ser	08-5578, 08-5923 (A118-like)
P7	Prophage	hypothetical protein LMHG_11046	FSL N1-017 (B054-like)

1 Selection of strains in which this insertion site is occupied by a mobile element or prophage. Between parenthesis resemblance to sequenced phages is indicated.

To further examine evolutionary changes in the accessory genome, we identified accessory loci that distinguish the two major lineages of *L. monocytogenes*, I and II (Table 5). Lineage II has significantly more distinguishing genes than lineage I (38 vs. 21; p = 0.03, chi-square test). Most functional categories from the enrichment analysis are represented within the lineage specific operons – both lineages have specific PTS operons (including transcriptional regulators) and cell-wall anchored proteins (including internalins). Furthermore, each lineage had a specific antimicrobial resistance-related operon/gene (Table 5; lineage I, anti-microbial peptide ABC-type transport system; lineage II, bacteriocin immunity protein). Despite inclusion of only two representatives of lineage III in our analysis (HCC23 and J2-071), this lineage showed a large degree of variation with respect to presence/absence of loci it from distinguishing lineages I and II, consistent with a previous array-based study [14].

**Table 5:** Accessory genome loci that distinguish lineages I and II.

Presence in Lineage^1^					
I	II	III	IV	Loci	Putative Function^2^
+	–	+	+	LMOf2365_0374	Internalin
+	–	+/−	+	LMOf2365_0413-0417	ABC-type antimicrobial peptide transport system, cell-wall-anchored protein
+	–	–	+	LMOf2365_0693-0694	Cell-wall-anchored proteins
+	–	+	+	LMOf2365_1131	Unknown
+	–	+	+	LMOf2365_1142-1143	Unknown
+	–	+/−	+/−	LMOf2365_1252-1254	Internalin, cell-wall-anchored
+	–	+/−	–	LMOf2365_1681-1683	N-acetylmuramic acid specific PTS
+	–	–	–	LMOf2365_2059	Regulatory protein
+	–	–	+	LMOf2365_2361	cAMP-binding protein
+	–	+	+	LMOf2365_2416	Internalin
+	–	–	–	LMOf2365_2638	Cell-wall-anchored protein
					
–	+	+/−	–	lmo0147	Unknown
–	+	–	+	lmo0171	Internalin, cell-wall-anchored
–	+	–	–	lmo0332	Unknown
–	+	+/−	–	lmo0341	Bacteriocin Immunity protein
–	+	+/−	–	lmo0421-0423	Cell division, lineage-specific thermal regulator protein, RNA polymerase factor sigma C
				lmo0525	Unknown
–	+	–	–	lmo0734-0739	PTS, putative pentose phosphate specific
–	+	+/−	–	lmo0749-750	putative regulatory protein
–	+	+	–	lmo0780	Unknown
–	+	–	–	lmo1060-1063	heavy metal associated two component response system and ABC transporter
–	+	+	+	lmo1125	Unknown
–	+	+	+	lmo1289	Internalin-like protein, Cell-wall-anchored
–	+	+	+	lmo1307	Unknown
–	+	–	–	lmo1968-1974	Creatinine amidohydrolase, KDPG and KHGaldolase, L-ascorbate specific PTS
–	+	+	–	lmo2169	Unknown
–	+	+/−	–	lmo2576	Putative collagen adhesion protein, cell-wall-anchored
–	+	+	+	lmo2644	Regulation of 1,3-beta-glucan synthase
–	+	–	–	lmo2686	Unknown
–	+	+/−	–	lmo2786-2788	glucose–glucoside (Glc) family PTS

1 + = present, – = absent, +/− = present in some strains.

2 The putative function is inferred from the initial gene annotation.

Presence/absence of orthologs in each of the four lineages is listed, as well as putative function and the locus identifier(s) in the reference genome, either F2365 (lineage I) or EGDe (lineage II).

### O-antigen Associated Genes seem to Follow a Serotype Specific Phylogenetic Pattern and show Several Instances of Horizontal Gene Transfer

**Figure 3:**
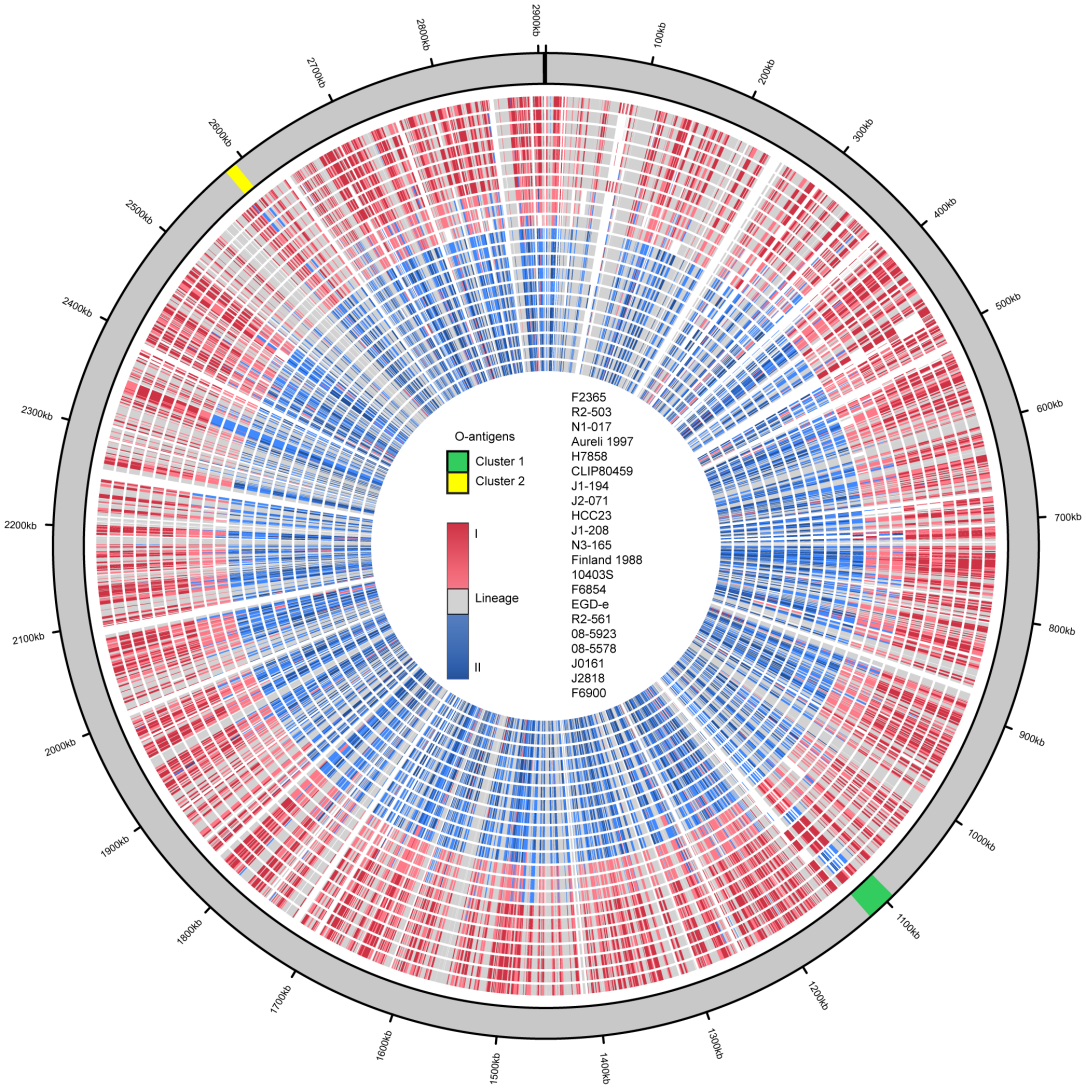
Clade membership plot of individual genes plotted against the genome of *L. monocytogenes* F2365. The order of genome rings is listed in the circle center, with F2365 being the outermost ring. The 7 outermost rings represent lineage I (serotype 4b and 1/2b), the next three rings represent lineage III and lineage IV strains (serotype 4a and 4c), and the last 11 rings represent lineage II strains (serotype 1/2a, 1/2c, and 3a). Clade membership of the individual genes is indicated by color; blue indicates lineage II, red indicates lineage I, and gray is unresolved membership. The two O-antigen gene clusters are highlighted in green and yellow. Genes in these clusters found in serotype 1/2b lineage I cluster phylogenetically with orthologs found in lineage II clade.

A phylogenetic approach to identify genes with evolutionary histories that deviate from the organismal phylogeny identified two gene clusters: (i) a cluster corresponding to lmo1074–1091 in *L. monocytogenes* EGD-e (cluster 1), and (ii) a cluster (cluster 2) corresponding to lmo2549-2558 in *L. monocytogenes* EGD-e (Fig. 3). These clusters are found in distinct regions of the genome; however, they both contain genes implicated in the biosynthesis of wall teichoic and lipoteichoic acids. Wall teichoic acids are associated with O-antigen variation [7], [59], [60] and because of this putative involvement, we will refer to these clusters as O-antigen clusters 1 and 2. For these clusters, the lineage I serotype 1/2b strains appear to have genes that are much more closely related to their orthologs in lineage II, which includes all the 1/2a and 3c strains, than to their orthologs in other lineage I strains (Fig.3). The phylogenetic distribution of serotype 1/2 related genes is incongruent with the organismal phylogeny (Fig. 1), and therefore horizontal transfer of these clusters from lineage II into lineage I could explain the occurrence of 1/2 serotypes in both lineages.

**Figure 4:**
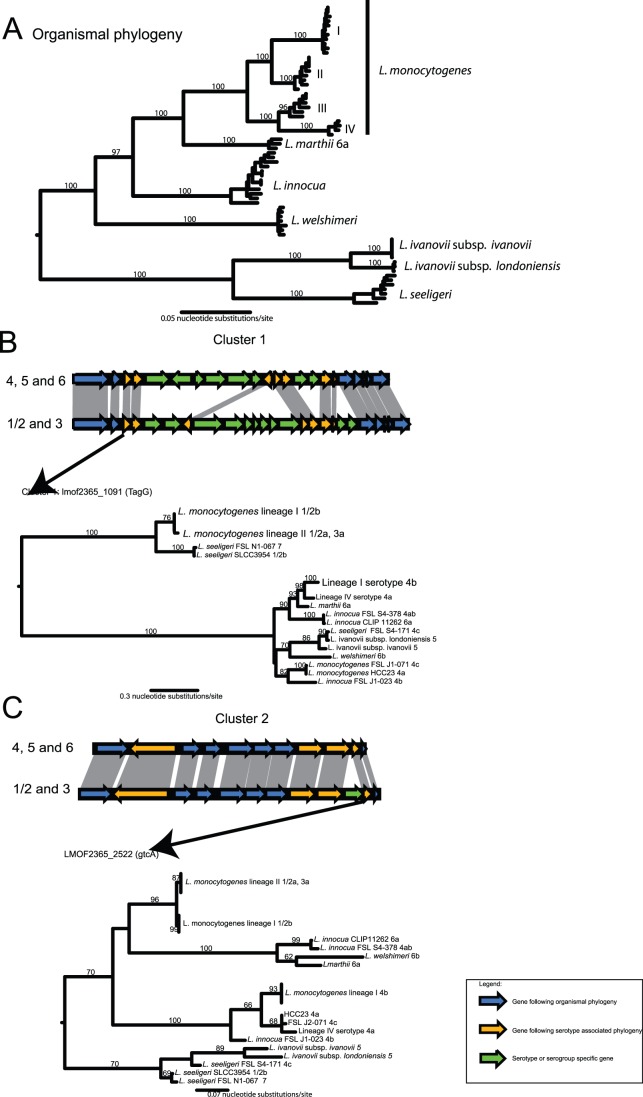
Synteny and gene-specific phylogenetic history of the two O-antigen specific gene clusters. The organismal phylogeny of the genus *Listeria* is shown in the upper panel (A), while the syntenic relationships of the two O-antigen gene clusters between the two major serotype divisions and the phylogenetic tree based on a representative serotype specific gene are shown in the two lower panels (B and C). Genes are colored by their phylogenetic histories: Serotype-specific genes (i.e., genes found only in specific serotypes) are colored green, while genes displaying an organismal phylogeny across the *Listera* genus are colored blue. Genes which follow a serotype-related phylogeny across *Listeria* are shown in orange. Values on the branches represent bootstrap values based on 100 bootstrap replicates. The organismal tree is based on a 10 locus multi-locus sequence analysis as described in Den Bakker et al. [52]. The topology of this tree is congruent with a tree based on the MLST scheme used in Ragon et al. [6].

Within a serotype (1/2 or 4, irrespective of alphabetical designation), all *L. monocytogenes* strains have largely the same gene content and order across both clusters (Fig. 4A, Figs. S1 and S2). Exceptions are a hypothetical protein (LMOf2365_1098 in strain F2365) in cluster 1 of lineage I serotype 4b strains and the lineage IV serotype 4 strain FSL J1-208. Between serotypes, O-antigen clusters 1 and 2 substantially differ in gene content (Fig. 4A, Fig. S1 and 2). The genomes of newly sequenced serotype 3a and 1/2c strains have identical gene content in the two serotype clusters as 1/2a strains, consistent with the phylogeny based on the concatenated alignments of the 2,086 core genes, which places the serotype 3a and 1/2c genomes among lineage II 1/2a strains (Fig. 1).

To determine if the phylogeny of O-antigen cluster genes is discordant with the organismal phylogeny across the entire *Listeria* genus we analyzed the gene content and synteny for both clusters in non-*L. monocytogenes Listeria* species for which genome sequences are available. In addition, we investigated other genes outside the two clusters which displayed a serotype related phylogenetic pattern, genes that were uniquely found within one serotype or the other, and genes that had been implicated in *L. monocytogenes* O-antigen variation in previous publications [20], [61], [62] (see supplemental Table S1 for key results). To aid in the analysis we also serotyped five additional *Listeria* strains (see Table 1). Gene content and gene order in cluster 1 was found to be highly similar between serotypes 1/2 (found in *L. monocytogenes* and *L. seeligeri),* 3 and 7 (found in *L. seeligeri* FSL N1-067 and in *L. monocytogenes* [58]), irrespective of the species in which the cluster was found (Fig. S1). While gene content and gene order in cluster 1 in serotypes 1/2, 3 and 7 are extremely similar among *L. monocytogenes* strains and even between species (*L. seeligeri* versus *L. monocytogenes*), we found this cluster to display subtle differences when serotypes 4, 5 and 6 were compared. Cluster 1 in *L. innocua* CLIP 11262 (serotype 6a) was found to be identical in gene content and gene order to *L. monocytogenes* serotype 4b and *L. monocytogenes* FSL J1-208 (serotype 4a). Gene content and gene order in cluster 1 of *L. welshimeri* SLCC5334 serotype 6b was found to be identical to *L. monocytogenes* serotype 4a (strain HCC23) and serotype 4c (strain FSL J2-071). We further found homologs of *gltA* and *gltB* in cluster 1 in *L. innocua* FSL J1-023 serotype 4b and in *L. ivanovii* serotype 5 (see Fig. S1). The *gltA-gltB* gene cassette was previously reported to be serotype 4b specific and involved in wall teichoic acid glycosylation [61]. This gene cassette is found in a region approximately 1.6 Mb removed from cluster 1 in *L. monocytogenes* serotype 4b isolates such as F2365 (LMOf2365_2740 and LMOf2365_2741).

To further probe the evolution of the two O-antigen clusters, we constructed gene phylogenies for genes, within these clusters, that had orthologs in both serotypes 1/2 and 4. Two phylogenetic patterns could be found among the shared genes in O-antigen cluster 1 (Fig. 4B): (i) a serotype-specific pattern, showing a clade consisting of serotypes 1/2, 3 and 7 and a clade consisting of serotypes 4, 5, and 6, (Fig. 4B, orange pattern; seven genes), and (ii) a pattern mirroring the organismal phylogeny of *Listeria* (Fig. 4B, blue pattern). The shared genes in cluster 2 also showed two distinct phylogenetic patterns (Fig. 4C): (i) a phylogenetic pattern reminiscent of the organismal phylogeny of *Listeria* and similar to that seen in cluster 1 (Fig. 4C, blue pattern), and (ii) a serotype-associated pattern for *L. monocytogenes, L. innocua, L. welshimeri* and *L. marthii*, but a non-serotype specific pattern for *L. seeligeri* and *L. ivanovii* (Fig. 4C, orange pattern; three genes). Cluster 1 genes with a serotype specific phylogenetic pattern were *tagG* (LMOf2365_1091) and *tagH* (LMOf2365_1092), an UTP-glucose-1-phosphate uridylyltransferase (homologous to *rfbA*: LMOf2365_1099), a glycosyl transferase (LMOf2365_1100), ribitol-5-phosphate cytidylyltransferase (LMOf2365_1101), *tagB* (CDP-glycerol:N-acetyl-beta-D-mannosaminyl-1,4-N-acetyl-D-glucosaminyldiphosphoundecaprenylglycerophosphotransferase: LMOf2365_1104) and a putative sorbitol dehydrogenase (LMOf2365_1105). Shared genes with a serotype specific phylogenetic pattern in cluster 2 were an autolysin (LMOf2365_2530), a gene annotated as UDP-N-acetylglucosamine 1-carboxyvinyltransferase (LMOf2365_2524), a transcription termination factor (LMOf2365_2523), and the cell wall teichoic acid glycosylation protein *GtcA* (LMOf2365_2522). Most of these shared genes with a serotype-associated phylogenetic pattern are homologous to genes implicated in basic functions in wall teichoic acid synthesis in other Firmicutes [63], [64], and in *L. monocytogenes* [60], [65], [66]. All wall teichoic acid associated genes that display a serotype-associated phylogenetic pattern show a high nucleotide divergence (e.g., 8.2–40%) between homologous genes of lineage I *L. monocytogenes* serotype 4b and *L. monocytogenes* 1/2b strains, while the nucleotide divergence between *L. monocytogenes* 1/2a (lineage II) and *L. monocytogenes* 1/2b (lineage I) strains is between 1.0 and 2.7%. The high nucleotide divergence suggests that 1/2- and 4- like serotypes predate the most common ancestor of *L. monocytogenes*. The fact that *L. monocytogenes* lineage III and IV, and closely related species such as *L. marthii* and *L. innocua* display 4 and 6 like serotypes, suggests that the most recent common ancestor of *L. monocytogenes* putatively was of serotype 4, and the 1/2-like serotypes were introduced, through horizontal gene transfer, in the ancestral population of *L. monocytogenes* lineage I and II. Alternatively both 1/2-like and 4-like serotypes could have been present in the ancestral *L. monocytogenes* population, and 4-like serotypes were subsequently lost in lineage II.

**Figure 5:**
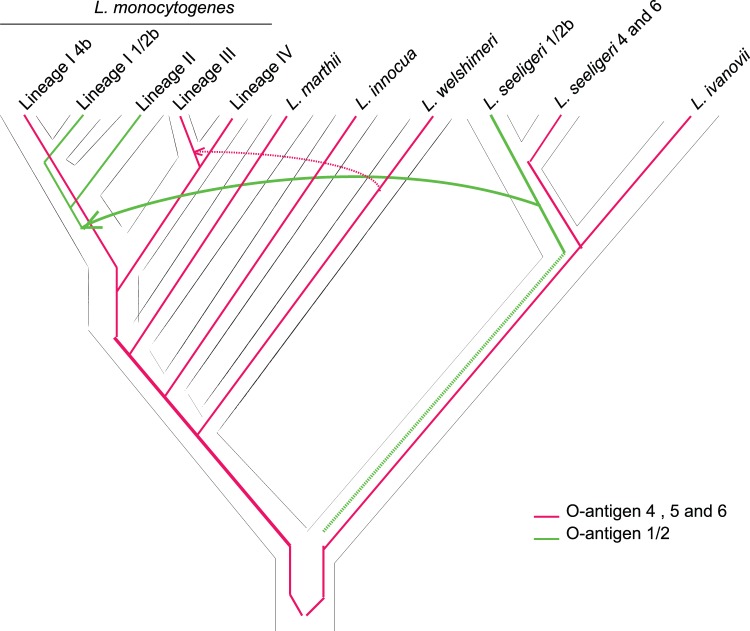
Phylogenetic reconstruction of serotype evolution in *Listeria*. Serotype 4 is shown in red while serotype 1/2 is shown in green. This construction suggests that serotype 1/2 genes were horizontally transferred from *L. seeligeri* to an ancestor of *L. monocytogenes* lineages I and II. The origin of the serotype 1/2 cluster is unclear, we hypothesize that this cluster putatively originated in the most recent common ancestor of the *L. seeligeri* and *L. ivanovii* clade (as indicated by the dashed line). Serotype 4 genes appear to be largely inherited by vertical descent, except for a lateral transfer of genes from *L. welshimeri* into some strains of *L. monocytogenes* lineage III (dotted red line).

To reconstruct the putative evolutionary history of serotypes in *L. monocytogenes* we reconciled the gene trees with serotype-specific patterns (Fig. 4B, red and orange patterns) with the organismal tree of the genus *Listeria* (similar to Fig. 4B, blue pattern) using the AnGST [53] and Mowgli [54] algorithms. Both algorithms simultaneously account for gene loss, gene duplications and horizontal gene transfer. The majority of the reconciliations for both cluster 1 genes (6/7 genes) and cluster 2 genes (3/3 genes) support a scenario in which horizontal gene transfer was responsible for the introduction of the 1/2 serotypes in the ancestral population of *L. monocytogenes* lineage I and II (Fig. 5). In the case of cluster 1, the putative donor of the genes encoding expression of the *L. monocytogenes* 1/2 serotypes was the ancestral population of *L. seeligeri*. Reconciliations of the cluster 2 genes suggest that the 1/2 serotypes arose once, either in the ancestral populations of *L. welshimeri* or *L. seeligeri.* The gene cluster was then transferred from these populations into the ancestral population of *L. monocytogenes* lineage I and II, and were subsequently lost in the donor populations.

In contrast to genes of the serotype 1/2 gene clusters, the serotype 4 O-antigen clusters followed a largely vertical descent through *Listeria* species (Fig. 5). The one exception to this mode of inheritance appears to be a replacement, in lineage III serotype 4a and 4c strains, of part of the ancestral O-antigen cluster 1 with a *L. welshimeri* type O-antigen cluster 1 through horizontal transfer. Horizontal transfer of the O-antigen cluster 1 into lineage III serotype 4a and 4c strains is further supported by the similarity in synteny of this cluster in both donor (*L. welshimeri* SLCC5334) and recipient (*L. monocytogenes* lineage III serotype 4a and 4c; see Fig. S1). All gene tree reconciliations support a most recent common ancestor of *L. monocytogenes,* which had serotype 4.

The phylogenetic patterns detailed above suggest the occurrence of homologous recombination within cluster 2 between *L. monocytogenes* donors and recipients. To test for homologous recombination and sequence tracts involved in these recombination events we used RAT [55] to detect putative breakpoints. We subjected sequences representing the entire cluster 2 (minus large indel regions) of *L. monocytogenes* serotypes 1/2b, lineage I 4b, *and* 1/2a to this analysis. The results of this analysis suggest that two sequence tracts within cluster 2 were putatively introduced into the lineage I serotype 1/2b strains from a lineage II serotype 1/2a donor. These tracts include (i) a tract encoding part of a homoserine dehydrogenase (lmo2547), the entire 50S ribosomal protein L31, gtcA, transcription termination factor Rho, UDP-N-acetylglucosamine 1-carboxyvinyltransferase, a hypothetical protein (lmo2555 homolog) and a glycosyl transferase, and (ii) a tract encoding an autolysin.

## Discussion

*L. monocytogenes* genomes are highly conserved and free of major genomic rearrangements even when compared to closely related *Listeria* species [67]. However, our work here suggests that this picture does not fully represent what appears to be an unappreciated property of this species; the Listerial genomes show evidence for uneven vulnerability to the gain of, or tolerance for, horizontal transfer based on position in the genome. The first 65° of the chromosome is enriched for accessory genes, while the last 295° is enriched for core genes; this genome compartmentalization is absent from the closely related bacteria such as *S. aureus* and *B. cereus*. There could be an adaptive value in such a behavior although the molecular mechanism responsible for this is unresolved. We also find a series of genes, which cluster phylogenetically according to serotype, but not according to the organismal phylogeny. The majority of these genes is organized in two gene clusters, and reconstruction of the putative evolutionary history of these clusters shows these genes have a complex evolutionary history, involving multiple instances of horizontal gene transfer.

The enrichment of the first 65° degrees of the genome for accessory genes can only be partly attributed to the eight hotspots recently described by Kuenne et al. [58] for this chromosomal region, as less than 25% of the accessory genome could be attributed to these hotspots. Overall, we found 38% of the accessory genome (prophage related genes not included) in the first 65° degrees of the genome. Kuenne et al. [58] used a strict definition of an insertion deletion hotspot (‘hotspots were defined by the localization of at least three non-homologous insertions between mutually conserved core genes’). We find that a large part of the accessory genome found in the first 65° degrees is found outside of the eight hotspots identified previously [58] and in the work reported here. We thus propose that this portion of the chromosome may be more accurately described as a "hot region" for the gain of horizontally acquired information.

The genome partitions we find in *L. monocytogenes* appear to stem from differences in selective pressures and different rates of gene insertion. The former is supported by the finding in *L. monocytogenes* genomes that core genes in the first 65° of the genome are under less purifying selection than genes in the last 295°, indicating that to some extent, the position of a gene within the genome may affect its rate of evolution regardless of whether the gene is part of the core or accessory genome. The size of intergenic regions is thought to be driven by, and reflective of, the balance between insertions and deletions [68]. The longer intergenic distances in accessory-rich region of the genome may reflect the dynamic nature of this region where the balance is tipped toward insertions of new accessory operons.

What molecular mechanism could account for one region becoming more prone to the accretion of foreign DNA? One possible explanation could involve systems that physically sequester regions of the genome. For example in *E. coli* the terminus region is physically and functionally gathered together through the action of the MatP protein that recognizes a series of sites (*matS*) in this region of the chromosome [69]. This region containing *matS* sites is constrained by another protein that seems to allow the terminus region to interact with the division machinery [70]). If a similar system worked in the first 65 degrees of the *L. monocytogenes* chromosome it could conceivable render this region differentially accessible for new DNA sequences that enter the cell. Interestingly the terminus region of the *E. coli* chromosome appears to evolve differently from the rest of the genome displaying lower rates of recombination without higher mutation rates [47].

Alternatively, as suggested previously for *E. coli* [47], one could also imagine a series of "domino" effects that follow the acquisition of a very large segment of DNA. If beneficial gene products were encoded in this DNA segment it could encourage maintenance of the new large DNA segment. However, genes on this same stretch of DNA that were under negative selection or were neutral would allow (if not encourage) the acquisition of more insertions. This entire new region would then be active for gain and loss of genes for a protracted period of time as deletions also occurred across the regions under negative or neutral selection. Eventually the original genes that allowed the new DNA to become fixed in the population would be unrecognizable from other core genes from the species, but the process of gaining more genetic information in the region and winnowing of the sequences under negative and neutral selection could occur over a much longer period of time. The net result would be a mosaic of core and accessory genes without any necessary association to mobile elements. Interestingly only one complete prophage can be found in the first 65° of the chromosome. Core genes found in this region may have only relatively recently become fixed in the population (or part of the core genome), which may explain why this region is more rapidly evolving compared to the rest of the chromosome.

Regardless of the mechanism that accounts for the regional effect suggested by our analyses, the compartmentalization of the *Listeria* chromosome into accessory gene rich and poor regions could provide an evolutionary risk management strategy analogous to one recently described in *E. coli*, where the chromosome is divided into mutational hot and cold spots [71]. In *E. coli*, mutational cold spots (regions with a lower mutation rate) coincide with highly expressed genes and genes under strong purifying selection, thereby reducing the risk of deleterious mutations in these regions [71].

Functional enrichment of transcriptional regulators, cell surface genes, and phosphotransferase systems in the accessory genome highlights the selective pressures faced by contemporary strains of *L. monocytogenes*. The complex regulation potentially required for networks of auxiliary or core genes to respond to these pressures may explain the abundance of transcription factors among the auxiliary genome. Enrichment of cell surface-related genes in the accessory genome of suggests that there is sustained selective pressure on *L. monocytogenes* to continually remodel the cell surface, playing a putative role in host specificity, host interactions, and the evasion of predators such as bacteriophages and protists in the non-host environment. Enrichment of cell surface-related genes in *L. monocytogenes* was also found in previous array based studies [14], [72]. These cell wall-enriched accessory genes include internalins, a class of genes that also encodes well characterized virulence factors such as internalin A, internalin B and internalin C [73]. The finding that phosphotransferase systems are enriched in the auxiliary genome suggests a selective pressure for *L. monocytogenes* to maintain a diverse repertoire of sugar transporters to cope with the diverse carbon sources in both hosts and the environment [74]. Another explanation for the diversification of phosphotransferase systems could involve interaction with other microbes, as it has been shown that certain phosphotransferase systems in *L. monocytogenes* are putative targets for bacteriocins [75]. A high diversity of phosphotransferase systems, combined with functional redundancy, may be a way to reduce bacteriocin sensitivity within host microbial communities.

While most genes in the *L. monocytogenes* genome follow a pattern of vertical descent, O-antigen associated genes and gene clusters seem to have distinct phylogenetic histories suggesting lateral transfers. A gene-by-gene gene-tree reconciliation approach suggests lateral transfer of O-antigen cluster 1 from a serotype 1/2 or 7 *L. seeligeri* ancestor into the serotype 4 *L. monocytogenes* ancestor. A putative change of function of O-antigen associated genes in cluster 2 in the *L. seeligeri* donor could explain the discrepancy between the phylogenetic patterns of cluster 1 and cluster 2 genes, where cluster 1 genes show a serotype specific pattern across *Listeria* species and cluster 2 genes only show a serotype-specific pattern within *L monocytogenes*. The fact that O-antigen cluster 2 genes in *L. seeligeri* 1/2b or 7 do not phylogenetically cluster according to serotype, suggests that genes in O-antigen cluster 1 are probably the most important determinants of O-antigen serotype. A break point analysis of *L. monocytogenes* cluster 2 suggests that Lineage I 1/2b serotype strains only recently acquired the serotype 1/2 gene fragments from Lineage II 1/2a donors. Further experimental work will be needed to clarify the role of cluster 1 and cluster 2 genes in serotype expression in different *L. monocytogenes* and *Listeria* species serotypes.

While serotype 1/2 was previously hypothesized to be the ancestral serotype in *L. monocytogenes* [6], our data support the alternative hypothesis, proposed here for the first time that 4-like serotypes were present in the ancestral population of *L. monocytogenes* lineages. This hypothesis seems to be supported by the observation that both lineage III and IV display 4-like serotypes, while the species most closely related to *L. monocytogenes* (i.e., *L. innocua* and *L. marthii*) also have 4-like serotypes. Based on the current data it is hard to refute the possibility that genes encoding serotype 1/2 expression (i.e., the clusters associated with this O-antigens) were introduced in the ancestor of both lineages I and II, and subsequently replaced by serotype 4 genes in a subset of lineage I. Additionally, while our gene tree reconciliations suggest that *L. seeligeri* was a donor of clusters 1/2, the reverse transfer cannot be excluded at this stage. More research on the function and evolution of these O-antigen related genes is necessary to unravel their complex evolutionary history and involvement in host-pathogen and bacteriophage interactions.

## Supporting Information

Figure S1 **Comparison of O-antigen cluster 1 in** ***L. monocytogenes*** **and closely related** ***Listeria*** **species.**
(EPS)

Figure S2 **Comparison of O-antigen cluster 2 in** ***L. monocytogenes*** **and closely related** ***Listeria*** **species.**
(PDF)

Table S1 **Summary of phylogenetic patterns found for wall teichoic and lipoteichoic acid associated genes in** ***Listeria*** **.**
(PDF)
