# Supplementary material for: Evolutionary Dynamics of the Accessory Genome of Listeria monocytogenes
Source: PLoS One. 2013 Jun 25;8(6):e67511. doi: 10.1371/journal.pone.0067511 (PMC3692452; doi:10.1371/journal.pone.0067511)
Supplement: Table S1 — Summary of phylogenetic patterns found for wall teichoic and lipoteichoic acid associated genes in Listeria . (PDF) [file pone.0067511.s003.pdf]

Table S1. Wall teichoic and lipotechoic acid associated genes and their phylogenetic patterns

locus id in EGD-e (lmo) or F2365 (LMOf2365)	organismal/serotype specific phylogeny ?	Gene symbol	divergence (% nucleotide identity)[1]	annotation
**genes with orthologues in all strains**				
LMOf2365_0119	serotype		2.7% (EGD-e 1/2a vs FSL R2-503 1/2b); 0.10204 (F2365 4b vs FSL R2-503 1/2b)	ArsR family transcriptional regulator
LMOf2365_0247	organismal	ispD		2-C-methyl-D-erythritol 4-phosphate cytidylyltransferase
LMOf2365_0678	organismal			conserved hypothetical protein
LMOf2365_0948	organismal	IspB		Lipoteichoic acid synthase LtaS Type Ia
LMOf2365_0979	organismal			Undecaprenyl-phosphate N-acetylglucosaminyl1-phosphate transferase
LMOf2365_0991	organismal	DltD		DltD protein for D-alanine esterification of lipoteichoic acid
LMOf2365_0992	organismal, lineage III/IV variant highly divergent	DltC		D-alanine--poly(phosphoribitol) ligase subunit 2
LMOf2365_0993	organismal	DltB		D-alanyl transfer protein DltB
LMOf2365_0994	organismal	DltA		D-alanine--poly(phosphoribitol) ligase subunit 1
LMOf2365_1091	serotype	TagG	1.1% (EGD-e 1/2a vs FSL R2-503); 23.7 % (F2365 vs FSL R2-503)	Teichoic acid translocation permease protein TagG
LMOf2365_1092	serotype	TagH	1.0% (EGD-e 1/2a vs FSL R2-503 1/2b); 21.7 % (F2365 4b vs FSL R2-503 1/2b)	Teichoic acid export ATP-binding protein TagH
LMOf2365_1093	serotype, highly divergent			autolysin (N-acetylmuramoyl-L-alanine amidase)
LMOf2365_1104	serotype	tagB	1.2 (EGD-e 1/2a vs FSL R2-503 1/2b); 40.6 (F2365 4b vs FSL R2-503 1/2b)	
LMOf2365_1646	organismal			Polysaccharide biosynthesis protein
LMOf2365_1647	organismal, lineage III/IV sister to I			polysaccharide biosynthesis family protein
LMOf2365_2492	organismal	tagO		undecaprenyl-phosphate N-acetylglucosaminyl1-phosphate transferase
LMOf2365_2494	organsimal	tagA		teichoic acid biosynthesis protein A (tagA)
LMOf2365_2522	serotype/organismal	GtcA	1.4% (EGD-e 1/2a vsFSL R2-5031/2b); 20.3% (F2365 4b vs FSL R2-503 1/2b)	cell wall teichoic acid glycosylation protein GtcA
LMOf2365_2523	serotype		1.6% (EGD-e 1/2a vs FSL R2-503 1/2b); 15.3% (F2365 4b vs FSL R2-5031/2b)	transcription termination factor Rho
LMOf2365_2524	serotype	murA-2	1.3% (EGD-e 1/2a vs FSL R2-503 1/2b); 16.3% (F2365 4b vs FSL R2-503 1/2b)	UDP-N-acetylglucosamine 1- carboxyvinyltransferase
LMOf2365_2525	organismal	lafC		conserved hypothetical protein
LMOf2365_2526	organismal	lafB		glycosyl transferase CpoA
LMOf2365_2527	organismal	lafA		glycosyl transferase
LMOf2365_2528	organismal			fructose-1,6-bisphosphate aldolase, class II
LMOf2365_2529	organismal, lineage III/IV variant highly divergent			putative lipid kinase
LMOf2365_2530			1.5% (EGD-e 1/2a vs FSL R2-503 1/2b); 8.2% (F2365 4b vs FSL R2-503 1/2b)	N-acetylmuramoyl-L-alanine amidase, family 2
**serotype 1/2 , 3 and 7 specific genes**				
lm01079				hypothetical protein
lmo1080		ggaB		minor teichoic acids biosynthesis protein GgaB
lmo1081				glucose-1-phosphate thymidylyltransferase
lmo1082				dTDP-4-dehydrorhamnose 3,5-epimerase
lmo1083		rfbB		dTDP-glucose 4,6-dehydratase
lmo1084				dTDP-4-dehydrorhamnose reductase
lmo1085		tagB homolog		CDP-ribitol:poly(ribitol phosphate) ribitol phosphotransferase
lmo1086				2-C-methyl-D-erythritol 4-phosphate cytidylyltransferase
lmo1087		tarJ-like		zinc-dependent alcohol dehydrogenase
lmo2550	1/2 specific, also in serotype 6	mtrA		
**serotype 4, 5, 6 specific genes**				
LMOf2365_0118				
LMOf2365_1094	4 specific, also in serotype 6			conserved hypothetical protein
LMOf2365_1095				glycosyl transferase family protein
LMOf2365_1096				hypothetical protein
LMOf2365_1097				glycosyl transferase family protein
LMOf2365_1098				hypothetical protein
LMOf2365_1099				UTP-glucose-1-phosphate uridylyltransferase
LMOf2365_1100				2-C-methyl-D-erythritol 4-phosphate cytidylyltransferase
LMOf2365_1101		tarJ-like		zinc-dependent alcohol dehydrogenase
LMOf2365_1102	4 specific, also in serotype 6	GgaA		galactosamine-containing minor teichoic acid biosynthesis protein GgaA
LMOf2365_1103	4 specific, also in serotype 6			CDP-glycerol:polyglycerol phosphateglycero-phosphotransferase
LMOf2365_2740	4 specific, in cluster 1 in *L. innocua* FSL J1-023 (*L. innocua* 4b) and *L. ivanovii* (serotype 5)	gltA		hypothetical protein
LMOf2365_2741	4 specific, in cluster 1 in *L. innocua* FSL J1-023 (*L. innocua* 4b) and *L. ivanovii* (serotype 5)	gltB		glycosyl transferase family protein
